# Prefrontal Consolidation and Compensation as a Function of Wearing Denture in Partially Edentulous Elderly Patients

**DOI:** 10.3389/fnagi.2019.00375

**Published:** 2020-01-31

**Authors:** Noriyuki Narita, Tomohiro Ishii, Sunao Iwaki, Kazunobu Kamiya, Masakazu Okubo, Takeshi Uchida, Ikuo Kantake, Koh Shibutani

**Affiliations:** ^1^Research Institute of Oral Science, Nihon University School of Dentistry at Matsudo, Matsudo, Japan; ^2^Department of Removable Prosthodontics, Nihon University School of Dentistry at Matsudo, Matsudo, Japan; ^3^Automotive Human Factors Research Center, National Institute of Advanced Industrial Science and Technology, Tsukuba, Japan; ^4^Dental Support Co. Ltd., Chiba, Japan; ^5^Department of Anesthesiology, Nihon University School of Dentistry at Matsudo, Matsudo, Japan

**Keywords:** prefrontal cortex, consolidation, compensation, chewing activity, denture wearing, elderly

## Abstract

**Background:**

The cognitive effects of wearing a denture are not well understood. This study was conducted to clarify the effects of denture use on prefrontal and chewing muscle activities, occlusal state, and subjective chewing ability in partially edentulous elderly individuals.

**Methods:**

A total of 16 partially edentulous patients were enrolled. Chewing-related prefrontal cortex and jaw muscle activities were simultaneously examined using a functional near-infrared spectroscopy (fNIRS) device and electromyography, under the conditions of unwearing, and wearing a denture. Occlusal state and masticatory score were also determined under both conditions. Using multiple linear regression analysis, associations between prefrontal and chewing activities with wearing were examined using change rates.

**Results:**

Chewing rhythmicity was maintained under both conditions. As compared with unwearing, the wearing condition was associated with improved prefrontal cortex and chewing muscle activities, occlusal state in regard to force and area, and masticatory score. Also, prefrontal activities were positively associated with burst duration and peak amplitude in masseter (Mm) and temporal muscle activities, as well as masticatory scores. In contrast, prefrontal activities were negatively associated with occlusal force.

**Conclusion:**

Wearing a denture induced a positive association between burst duration and peak amplitude in Mm and temporal muscle activities and prefrontal activity, which may indicate a parallel consolidation of prefrontal cortex and rhythmical chewing activities, as well as masticatory scores. On the other hand, denture use induced a negative association of occlusal force with prefrontal activities, which might suggest that prefrontal compensative associations for the physiocognitive acquisition depended on biomechanical efficacy gained by wearing a denture.

## Introduction

Epidemiologic studies have suggested associations between cognitive ability and oral conditions, such as number of teeth (Luo et al., 2015; Li et al., 2017; Oh et al., 2018) and dental occlusion (Ono et al., 2010; Franco et al., 2012; Takeuchi et al., 2015). Furthermore, occlusal force (Takeshita et al., 2016; Ikebe et al., 2018) and chewing ability have been shown to have effects on cognitive ability (Chen et al., 2015; Natalie et al., 2015; Seraj et al., 2017). Oral reconstruction by means of wearing a denture may induce cortical activation in prefrontal, sensorimotor, and sensory association cortices during chewing performance (Kimoto et al., 2011; Kamiya et al., 2016) and might also contribute to antiaging cognitive activation in the prefrontal cortex of elderly edentulous patients (Banu et al., 2016).

Denture use is considered to have effects on not only physical activity but also cognitive demands in aged individuals (Cerutti-Kopplin et al., 2015; Shin et al., 2019). Thus, a well-fitting denture may help to maintain cognitive ability and prevent its decline otherwise caused by tooth loss in elderly edentulous patients. On the other hand, in individuals with tooth loss who do not wear a denture, cognitive decline might be accelerated with age.

The association of a higher cognitive node with biomechanical state caused by wearing a denture remains unknown, though a functional association of prefrontal activity with chewing ability in elderly patients has been proposed (Lin et al., 2016). The present study was conducted to clarify the biomechanical efficacy of wearing a denture based on the associations of prefrontal cortex activities with physical chewing activities in partially edentulous elderly patients.

In addition to investigations of chewing-related prefrontal participation under wearing a denture and tooth loss conditions, studies that used a cognitive task demanding consolidation or compensation by prefrontal cortex activities during physical task performances have been presented (Bhambhani et al., 2006; Tsujii et al., 2013; Moriya et al., 2016; Mirelman et al., 2017; Hawkins et al., 2018). Thus, the elucidation of the relationships between consolidated or compensated prefrontal activities and physical chewing activities in partially edentulous elderly patients would help with evaluation of the efficacy and quality of wearing a denture in the process of oral neurorehabilitation. The present is the first known study to explore the possibility of neurorehabilitation by use of a denture in partially edentulous elderly subjects.

Functional near-infrared spectroscopy has been used to reveal the hemodynamic response to stimulus-induced cortical activation in order to evaluate the neurobiology in exercise and cognition (Bhambhani et al., 2006; Tsujii et al., 2013; Moriya et al., 2016), and chewing cognition was also examined by fNIRS in both young and aged subjects (Hasegawa et al., 2013; Kamiya et al., 2016; Yokoyama et al., 2017) as well as psychiatric patients with persistent occlusal dysesthesia (Narita et al., 2019). fNIRS was used in the present investigation, as it is considered suitable for examinations of prefrontal and chewing activities with and without wearing a denture in clinical situations.

## Materials and Methods

### Participants

**FIGURE 1:**
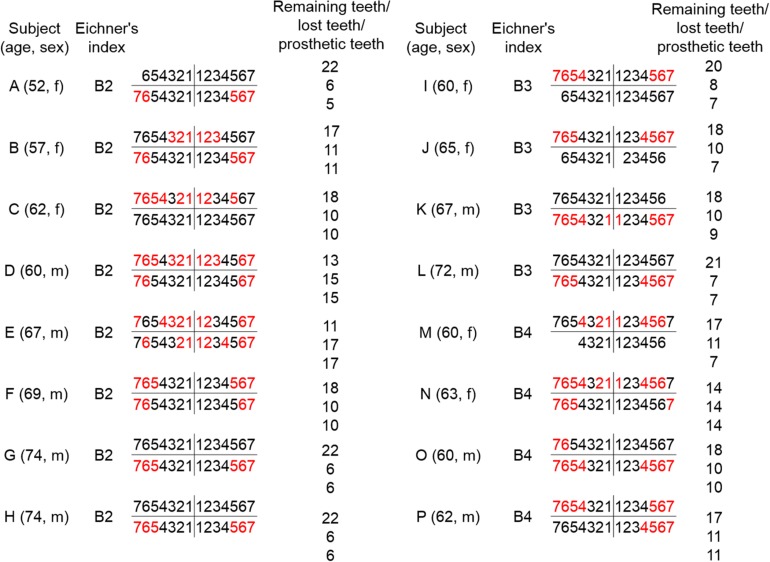
Age, gender, and dental state in partially edentulous patients. Age, gender, and dental state for each of the 16 partially edentulous patients **(A–P)** are shown. Subjects **A–H**, **I–L**, and **M–P** were classified as B2, B3, and B4, respectively, according to Eichner’s intermaxillary tooth contact classification. The remaining teeth are indicated in black and prosthetic teeth in red.

A total of 16 partially edentulous patients {nine males and seven females; mean age 64.0 ± 6.2 years [mean ± standard deviation (SD)]} undergoing treatments at the Prosthodontics Department of Nihon University School of Dentistry at Matsudo Hospital were enrolled in this study. The G^∗^Power 3 software package (non-commercial program downloaded from the University of Dusseldorf, Germany) (Faul et al., 2007) was used to determine the sample size, which established parameters with a significance level of 0.05, statistical power of 0.8, and effect size of 0.25 (medium effect). From those findings, the number of subjects needed to detect significant differences in this study was concluded to be 16. Statistical results were obtained with the power of the performed test at α = 0.050. The mean (±SD) numbers of the remaining, lost, and prosthetic teeth in the present patients were 17.9 ± 3.2, 10.2 ± 3.2, and 9.8 ± 3.4, respectively (Figure 1). Based on Eichner’s intermaxillary tooth contact classification (Eichner, 1955), we divided the subjects into three groups (B2, *n* = 8; B3, *n* = 4; and B4, *n* = 4), with B2 indicating two supporting zones, B3 indicating one supporting zone, and B4 indicating no supporting zones (Figure 1). None of the patients noted prosthesis conditions, such as discomfort or pain, or had difficulties with chewing performance and symptoms of temporomandibular joint or masticatory muscle dysfunction such as jaw pain or jaw movement difficulties. Individual patterns of tooth loss in each patient, as well as details of their tooth defects and partial denture-wearing condition, are presented in Figure 1. This study was conducted in accordance with the Declaration of Helsinki and approved by the Ethics Committee of Nihon University School of Dentistry at Matsudo (EC 14-13-010-1). Prior to beginning the examinations, all patients provided written informed consent for participation.

### Experimental Procedures

Each patient received sufficient prosthodontic treatment in the form of conventional removable partial denture prostheses during the 3 months prior to beginning the study. Masticatory score, occlusal state, and jaw muscle and prefrontal activities during chewing were determined to elucidate changes in chewing ability, occlusal force or occlusal area, jaw muscle activity, and prefrontal activity during chewing caused by wearing a denture. Measurements were conducted under the conditions of rest (Rest), without use of a denture (Unwearing), and while wearing a denture (Wearing). With the patient comfortably seated in a quiet room, masticatory muscle and prefrontal activities were recorded during gum chewing simultaneously using fNIRS and EMG devices under the Unwearing and Wearing conditions, while those were also recorded at Rest without gum chewing. Chewing ability and occlusal force and area were evaluated using the food intake questionnaire and the pressure-sensitive sheets under the Unwearing and Wearing conditions, respectively (Hirai et al., 1994; Miura et al., 1998).

### Chewing Task

Chewing sessions consisted of four different chewing performances, as follows: (1) chewing on the left side under Unwearing, (2) chewing on the left side under Wearing, (3) chewing on the right side under Unwearing, and (4) chewing on the right side under Wearing. The four chewing sessions were performed randomly to avoid the influence of task sequence on the results. Each session included five chewing trials, with the trial conducted for 10 s with gum chewing and separated from the succeeding chewing trial by a 40-s rest phase. One piece of chewing gum (Freezone, Lotte Corporation, Japan) was used for the chewing task under both the Unwearing and Wearing conditions. The patients were instructed to be quiet until given a verbal cue (pre-task period), and then a verbal cue was given to start gum chewing for 10 s (task period) until a verbal cue to stop chewing was given, after which they were instructed to remain quiet for 20 s (post-task period). In addition, the patients performed a Rest session without a chewing task and were asked to remain quiet during that time.

### Masticatory Score

Masticatory score, determined based on results of self-assessed chewing ability under the Unwearing and Wearing conditions, was used to evaluate subjective chewing ability (Hirai et al., 1994; Miura et al., 1998). Each patient rated their ability to chew 35 different food items according to the following scale: 2, can be eaten easily; 1, can be eaten with difficulty; 0, cannot be eaten (Hirai et al., 1994; Miura et al., 1998).

### Occlusal State

In order to evaluate occlusal state, bilateral maximal occlusal force, occlusal area, average pressure, and maximum pressure were examined under the Unwearing and Wearing conditions using 97-μm-thick pressure-sensitive sheets (Dental Prescale 50H R-type, Fuji Film Co., Tokyo, Japan), with the patients asked to perform maximal clenching in an intercuspal position after placement of the sheet. Values for those parameters were obtained by use of analytic equipment (Occluzer FPD703, Fuji Film Co., Tokyo, Japan).

### Masticatory Muscle EMG Activity

Masticatory muscle EMG activities from the left and right masseter (Mm, jaw closing), anterior temporal (Ta, jaw closing), and anterior digastric (AD, jaw opening) muscles were recorded using a multichannel EMG device (Polygraph Bioelectric Amplifier 1253A, San-ei MED, Tokyo, Japan) during chewing under the Unwearing and Wearing conditions. Electrodes were positioned bilaterally on the center of the muscle parallel with the direction of the muscle fibers and an interelectrode distance of 20 mm, with a ground electrode attached to the left earlobe. Amplified EMG signals were digitized with 16-bit resolution using an A/D converter [APA16-32/2(OB) F, CONTEC, Tokyo, Japan] and then downloaded to a personal computer at a sampling rate of 1 kHz.

### Prefrontal fNIRS During Chewing Performance

A 22-channel fNIRS device (ETG-100, Hitachi Medical Co., Chiba, Japan), which utilizes near-infrared light at wavelengths of 780 and 830 nm (Strangman et al., 2002), was used to assess the activity of the prefrontal cortex during chewing performance. Each fNIRS probe was fitted with a 3 × 5 thermoplastic shell and placed in the prefrontal region, with the bottom lines of the probes set according to Fp1 and Fp2, with referral to the international 10–20 system (Klem et al., 1999). Change in oxygenated hemoglobin concentration [(oxy-Hb)] was used as an indicator of change of brain activity, as previously validated (Hoshi et al., 2001). Change in [oxy-Hb] has been shown to have a strong correlation with blood-oxygenation-level-dependent signals measured by fMRI (Toronov et al., 2001). The sampling interval was 0.1 s. During the measurements, the patients were instructed to open their eyes and gaze at a point in front of them. Each trial was repeated five times, and [oxy-Hb] was measured during chewing performance on the left and right sides under the Unwearing and Wearing conditions, as well as during Rest. The obtained [oxy-Hb] values were averaged using the “integral mode” of the ETG-100 software. Measurement baselines were corrected using linear fitting (Sawa et al., 2012), which was performed by connecting the pre-task baseline (mean of final 20 s of the pre-task period) with the post-task baseline (mean of final 20 s of the post-task period).

### Anatomic Localization of fNIRS Channels

**FIGURE 2:**
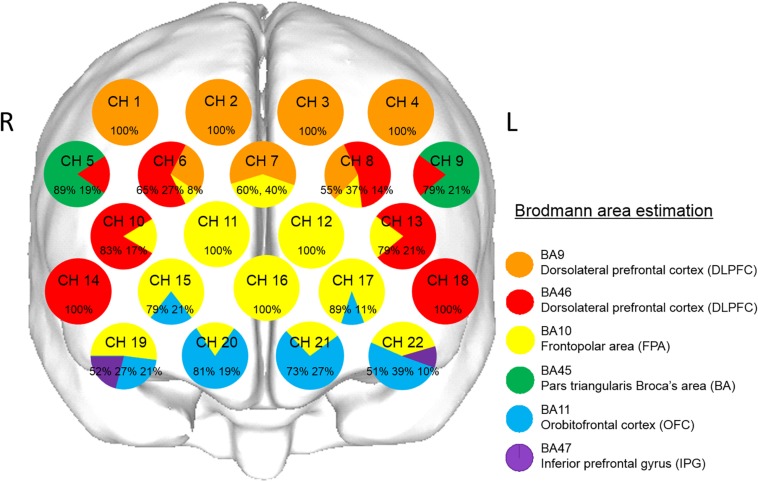
Anatomical identification of near-infrared spectroscopy channels. The coordinates for all probe and anatomical landmark positions (Nz, Iz, A1, A2, and Cz) were obtained using a three-dimensional digitizer. Probabilistic registration was used to transcribe the measuring points for each subject according to the protocol of the Montreal Neurological Institute, and those points were projected onto the cortical surface. Anatomical localization was identified using the Platform for Optical Topography Analysis Tools, with reference to the Automated Anatomical Labeling system. Orange, red, yellow, green, blue, and purple represent DLPFC (BA9), DLPFC (BA46), FPA (BA10), BA (BA45), OFC (BA11), and IPG (BA47), respectively. Each circle corresponds to a channel, and the pie chart within each circle shows the percentages of areas in that channel.

The coordinates for all probe and anatomical landmark (Nz, Iz, A1, A2, and Cz) positions were obtained using a three-dimensional digitizer (3SPACE ISOTRAK2, Polhemus, United States) and then transcribed into Montreal Neurological Institute standard brain space (Collins et al., 1994; Brett et al., 2002) using probabilistic registration (Okamoto and Dan, 2005; Singh et al., 2005). The probe positions were projected onto the cortical surface, with Platform for Optical Topography Analysis Tools (POTATo, Hitachi, Japan) used to identify the anatomic localization corresponding to each probe coordinate, with reference to the Brodmann area (Tzourio-Mazoyer et al., 2002; Okamoto and Dan, 2005; Singh et al., 2005; Figure 2). Representative anatomical identification of the fNIRS channels is shown in Figure 2. Those channels were localized in the dorsolateral prefrontal cortex (DLPFC, BA9 and BA46), frontopolar area (FPA, BA10), pars triangularis Broca’s area (BA, BA45), orbitofrontal cortex (OFC, BA11), and inferior prefrontal gyrus (IPG, BA47).

### Data and Statistical Analyses

#### Averaging of Masticatory Muscle and Prefrontal Cortex Activities During Chewing Performance

Masticatory muscle activities, such as number of chewing strokes, cycle duration, burst duration, inter-burst duration, area, mean amplitude, and peak amplitude, were averaged using data obtained for right- and left-side chewing. Those results showed no significant differences in regard to the number of remaining teeth, chewing muscle activities, or prefrontal [oxy-Hb] values between right- and left-side chewing under the Unwearing and Wearing conditions (Supplementary Material).

#### Comparing Masticatory Score, Masticatory Muscle Activities, Occlusal Force, Occlusal Area, Average Pressure, and Max Pressure Between Unwearing and Wearing Conditions

A paired *t*-test or Wilcoxon’s signed rank test was used for comparing masticatory score, masticatory muscle activities, occlusal force, occlusal area, average pressure, and max pressure between Unwearing and Wearing conditions, depending on normality test results.

#### Comparing Prefrontal [oxy-Hb] Values Between the Rest, Unwearing, and Wearing Conditions

Furthermore, averaged data for prefrontal [oxy-Hb] were accumulated during chewing periods (10 s) from all 22 channels under [oxy-Hb] values. One-way repeated-measures ANOVA and Tukey’s test or Friedman’s repeated-measures ANOVA on ranks and Dunn’s test were applied for analyzing prefrontal [oxy-Hb] values obtained during chewing periods in order to compare between the Rest, Unwearing, and Wearing conditions, depending on normality test results.

#### Multiple Linear Regression Analysis Dependent Variable (Prefrontal Activities) and Independent Variables (Occlusal States and Chewing Activities)

Change rate (difference between Wearing and Unwearing values, divided by the Unwearing value) was used to standardize the prefrontal [oxy-Hb] values as dependent variables and to standardize values for masticatory score, burst duration, peak amplitude, and occlusal force as independent variables.

Multiple linear regression was performed using a model corresponding to each variable, with the following equation:

Y=a⁢x1⁢1+a⁢x2⁢2+a⁢x3⁢3+a⁢x4⁢4+c

where *Y* is the dependent variable; *x*1, *x*2, *x*3, and *x*4 are independent variables; *a*_1_, *a*_2_, *a*_3_, and *a*_4_ are regression coefficients; and *c* is the error term with an *N*(0, σ^2^) distribution.

When performing multiple regression analysis, an important assumption is that the independent variables are truly independent of each other; i.e., there is no relationship among any of the independent variables used to estimate ordinary least squares. However, in some applications of regression, independent variables are in fact related to each other, which is termed a multicollinearity problem (Chatterjee and Hadi, 2006). For the present study, correlation analysis by Pearson’s correlation coefficient was used to check for a multicollinearity problem among the independent variables. Variables with a correlation coefficient (*r*) of ≥0.5 were considered to be strongly correlated with each other. To avoid multicollinearity, separate models were built using variables with lower levels of correlation with each other (*r* < 0.5). Evaluations of the multivariate models were conducted using coefficients of determination (*r*). Any intercorrelation between the four independent variables, change rates in the masticatory score, occlusal force, burst duration, and peak amplitude of Mm or Ta between the Unwearing and Wearing conditions, was subjected to bivariate correlation coefficient evaluation. In the present study, a correlation was found between the muscle activities of Mm or Ta; thus, they were considered separately in the model.

The statistical software package SigmaPlot 12.5 (Systat Software Inc., CA, United States) was used for all analyses, and *p* values less than 0.05 were considered to be statistically significant. Results are expressed as the Mean ± SD, *p* value, or power of the performed test.

## Results

### Effects of Wearing a Denture on Masticatory Score, Occlusal State, and Masticatory Muscle EMG Activities

#### Masticatory Score

Masticatory score under the Wearing condition were significantly (*p* < 0.001, paired *t*-test, power of performed two-tailed test with α = 0.050: 1.000) increased as compared with those under the Unwearing condition (Table 1).

**TABLE 1:** Masticatory scores under Unwearing and Wearing conditions.

	**Unwearing**		**Wearing**		***P* value**
	**Mean**	***SD***	**Mean**	***SD***	
Masticatory score	48.0	23.1	81.3	9.5	<0.001***

***Significant difference between Unwearing and Wearing conditions (paired *t*-test, *p* < 0.001).

#### Occlusal State

Occlusal force under the Wearing condition was significantly (*p* < 0.001, paired *t*-test, power of performed two-tailed test with α = 0.050: 1.000) increased as compared with that under the Unwearing condition (Table 2). Additionally, the occlusal contact area under the Wearing condition was significantly (*p* < 0.001, paired *t*-test, power of performed two-tailed test with α = 0.050: 1.000) increased as compared with that under the Unwearing condition (Table 2). Average and max pressure were not significantly (paired *t*-test and Wilcoxon’s signed rank test, respectively) different between these conditions (Table 2).

**TABLE 2:** Occlusal states under the Unwearing and Wearing conditions.

**Occlusal states**	**Unwearing**		**Wearing**		***p* value**
	**Mean**	***SD***	**Mean**	***SD***	
Occlusal force (N)	289.3	105.9	620.0	132.5	<0.001***
Occlusal contact area (mm^2^)	6.6	2.6	14.2	3.7	<0.001***
Average pressure (MPa)	43.3	5.7	42.9	5.5	0.89
Maximum pressure (MPa)	100.6	14.3	104.6	12.3	**0.130**

***Significant difference between the Unwearing and Wearing conditions (paired *t*-test, *p* < 0.001). Paired *t*-test is indicated in normal font, and Wilcoxon’s signed rank test is indicated in bold font.

#### Masticatory Muscle EMG Activities During Chewing Performance

The number of chewing strokes for Mm under the Wearing condition was not significantly (paired *t*-test) different as compared to that under the Unwearing condition, and cycle duration for AD EMG activity was also not significantly (paired *t*-test) different between the conditions (Table 3). Burst duration for Mm EMG activity under the Wearing was significantly (*p* = 0.010, paired *t*-test, power of performed two-tailed test with α = 0.050: 0.615) elongated as compared with that under the Unwearing condition. Under the Wearing condition, burst duration for Ta EMG activity was not significantly (paired *t*-test) different with that under Unwearing condition, and the inter-burst duration for Mm and Ta EMG activities was also not significantly (paired *t*-test) different between these conditions (Table 3). In contrast, the areas for Mm and Ta under the Wearing condition were significantly (*p* ≤ 0.001, Wilcoxon’s signed rank test) increased, and the mean amplitude for Mm EMG activity under the Wearing condition was also significantly (*p* = 0.001, Wilcoxon’s signed rank test) increased as compared with that under the Unwearing condition (Table 3). Also, the mean amplitude for Ta EMG activity under the Wearing condition was significantly (*p* = 0.044, paired *t*-test, power of performed two-tailed test with α = 0.050: 0.539) increased as compared with that under the Unwearing condition, while the peak amplitude for Mm EMG activity was not significantly (paired *t*-test) different between these conditions (Table 3). Finally, peak amplitude for Ta EMG activity under the Wearing condition was significantly (*p* = 0.012, paired *t*-test, power of performed two-tailed test with α = 0.050: 0.759) increased as compared with that under the Unwearing condition (Table 3).

**TABLE 3:** Masticatory muscle EMG activities under the Unwearing and Wearing conditions.

**Masticatory muscle EMG activities**		**Unwearing**		**Wearing**		
		**Mean**	**SD**	**Mean**	**SD**	***p* value**
Number of chewing strokes	Mm	63.28	13.44	65.16	13.34	0.55
Cycle duration (ms)	AD	837.28	123.56	838.71	173.34	0.55
Burst duration (ms)	Mm	306.54	87.03	344.67	72.93	0.03*
	Ta	282.19	67.71	297.80	59.95	0.49
Inter-burst duration (ms)	Mm	535.75	102.93	503.19	121.81	0.96
	Ta	561.60	100.59	545.19	132.23	0.26
Area (mV⋅s)	Mm	0.01	0.01	0.02	0.01	< 0.001†††
	Ta	0.01	0.00	0.02	0.01	< 0.001†††
Mean amplitude (mV)	Mm	0.04	0.03	0.06	0.04	0.001†††
	Ta	0.04	0.02	0.05	0.03	0.04*
Peak amplitude (mV)	Mm	0.10	0.07	0.13	0.08	0.34
	Ta	0.07	0.04	0.09	0.05	0.01*

*Significant difference between the Unwearing and Wearing conditions (paired *t*-test, *p* < 0.05). **†⁣†⁣†**Significant difference between the Unwearing and Wearing conditions (Wilcoxon’s signed rank test, *p* < 0.001).

### Grand Average Waveforms for Changes in Prefrontal [oxy-Hb]

**FIGURE 3:**
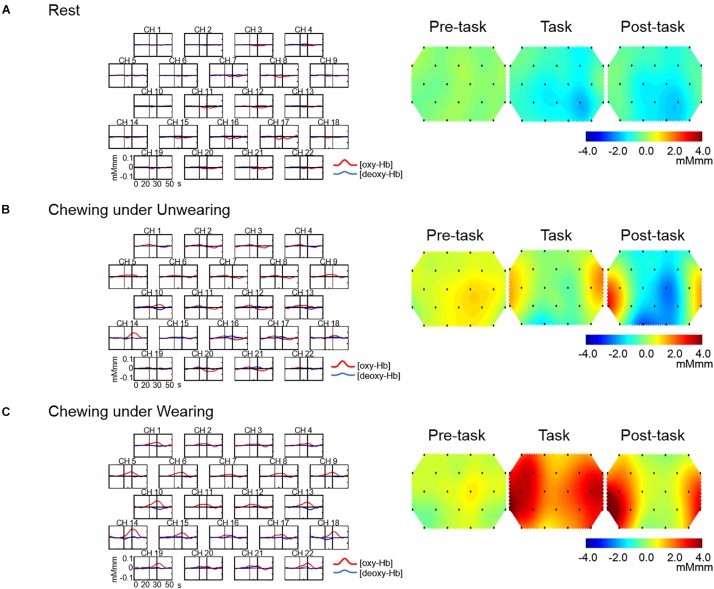
Grand average waveforms for oxygenated hemoglobin concentration [(oxy-Hb)] and deoxygenated hemoglobin concentration [(deoxy-Hb)] and topographical maps showing changes in [oxy-Hb] during Rest and chewing under Unwearing and Wearing conditions. On the left are grand average changes in [oxy-Hb] (red line) and [deoxy-Hb] (blue line) for each of the 22 measurement channels during the Rest condition **(A)**, chewing under the Unwearing **(B)**, and Wearing **(C)** conditions. The *x*-axis indicates time (s) and the *y*-axis hemodynamic change (mMmm). Vertical lines at 20 and 30 s indicate the start and end of the 10-s chewing period. On the right are 10-s topographical maps showing changes in [oxy-Hb] preceding (pre-task), during (task), and following (post-task) rest **(A)**, chewing under the Unwearing **(B)**, and Wearing **(C)** conditions. There was little change in [oxy-Hb] throughout the Rest session **(A)**, while there was a slight increase during the chewing period under the Unwearing **(B)** condition and marked increases during chewing and post-chewing under the Wearing **(C)** condition.

The grand averaged waveforms for changes in prefrontal [oxy-Hb] and prefrontal deoxygenated hemoglobin concentration [(deoxy-Hb)] under the Rest, Unwearing, and Wearing conditions are shown in Figures 3A–C, respectively. Unwearing resulted in slight changes in prefrontal [oxy-Hb] during the chewing period as compared with Rest (Figures 3A,B). In contrast, under the Wearing condition, marked and broad increases in prefrontal [oxy-Hb] were found during the task period, as compared to Rest and Unwearing conditions (Figures 3A–C).

### Topographical Changes in Prefrontal [oxy-Hb] During 10-s Task Periods Under Rest, Unwearing, and Wearing Conditions

Topographical maps for changes in prefrontal [oxy-Hb] in the pre-task, task, and post-task periods under the Rest, Unwearing, and Wearing conditions are shown in Figures 3A–C, respectively. An equivocal change in prefrontal [oxy-Hb] throughout the Rest condition was noted (Figure 3A), while slight changes in prefrontal [oxy-Hb] presented during chewing under the Unwearing condition in the pre-task, task, and post-task periods, as compared with the Rest condition (Figures 3A,B). In contrast, a marked increase in prefrontal [oxy-Hb] during the chewing session under the Wearing condition in the task and post-task periods as compared to the Rest and Unwearing conditions was noted (Figures 3A–C).

### Accumulated Prefrontal [oxy-Hb] During the Task Period

Prefrontal [oxy-Hb] in the task periods under the Rest, Unwearing, and Wearing conditions was analyzed using one-way repeated-measures ANOVA and Friedman’s repeated-measures ANOVA on ranks in R-DLPFC (CH 1, power of performed test with α = 0.050: 0.963), R-DLPFC (CH 2), L-DLPFC (CH 3 and CH 4), R-BA/DLPFC (CH 5, power of performed test with α = 0.050: 1.000), R-DLPFC/FPA (CH 6), RL-DLPFC/FPA (CH 7, power of performed test with α = 0.050: 0.760), L-DLPFC/FPA (CH 8, power of performed test with α = 0.050: 0.981), L-BA/DLPFC (CH 9, power of performed test with α = 0.050: 0.943), R-DLPFC/FPA (CH 10), R-FPA (CH 11), L-FPA (CH 12), L-DLPFC/FPA (CH 13, power of performed test with α = 0.050: 0.972), R-DLPFC (CH 14, power of performed test with α = 0.050: 0.893), R-FPA/OFC (CH 15), RL-FPA (CH 16), L-FPA/OFC (CH 17), L-DLPFC (CH 18), R-FPA/OFC/IPG (CH 19), R-OFC/FPA (CH 20), L-OFC/FPA (CH 21), and L-OFC/FPA/IPG (CH 22, power of performed test with α = 0.050: 0.717).

### Unwearing as Compared With Rest

**FIGURE 4:**
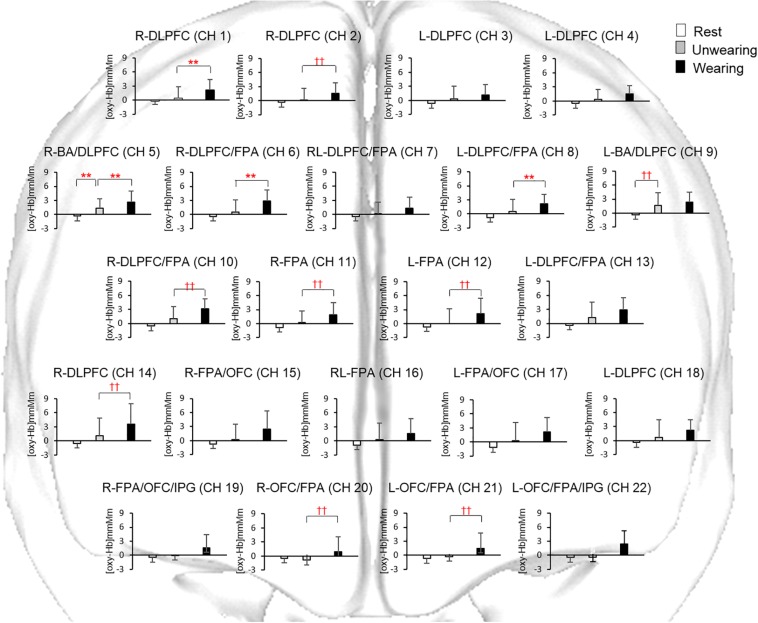
Accumulated oxygenated hemoglobin concentration [(oxy-Hb)] under the Rest condition and during chewing under the Unwearing and Wearing conditions. Statistical analyses were performed using one-way repeated measures ANOVA and pairwise multiple comparison procedures of Tukey’s test, as well as Dunn’s test. **Significant difference between Rest and Unwearing, and between Unwearing and Wearing conditions (one-way repeated-measures ANOVA and Tukey’s test, *p* < 0.01). ^††^Significant difference between Rest and Unwearing, and between Unwearing and Wearing conditions (Friedman’s repeated-measures ANOVA on ranks and Dunn’s test, *p* < 0.01). Accumulated [oxy-Hb] under Unwearing significantly increased for BA/DLPFC (CH 5, 9) as compared to Rest condition. Accumulated [oxy-Hb] under Wearing also significantly increased for DLPFC (CH 1, 2, 14), BA/DLPFC (CH 5), DLPFC/FPA (CH 6, 8, 10), FPA (CH11, 12), and FPA/OFC (CH 20, 21) as compared to Unwearing condition.

During the chewing session under the Unwearing condition, a significant (*p* < 0.01, one-way repeated-measures ANOVA and Tukey’s test, Friedman’s repeated-measures ANOVA on ranks, and Dunn’s test) increase in accumulated prefrontal [oxy-Hb] in RL-DLPFC/BA (CH 5, 9) was noted during the task period as compared with that under the Rest condition (Figure 4).

### Wearing as Compared With Rest

During the chewing session under the Wearing condition, significant (*p* < 0.05, one-way repeated-measures ANOVA and Tukey’s test and Dunn’s test) increases in accumulated prefrontal [oxy-Hb] in R-DLPFC (CH 1, 2, 14), L-DLPFC (CH 3, 4, 18), R-DLPFC/FPA (CH 6, 10), RL-DLPFC/FPA (CH 7), L-DLPFC/FPA (CH 8), L-DLPFC/FPA (CH 13), R-DLPFC/BA (CH 5), L-DLPFC/BA (CH 9), R-FPA (CH 11), L-FPA (CH 12), RL-FPA (CH 16), R-FPA/OFC (CH 15), L-FPA/OFC (CH 17), R-OFC/FPA (CH 20), R-OFC/FPA (CH 21), R-FPA/OFC/IPG (CH 19), and L-FPA/OFC/IPG (CH 22) were seen, as compared with that under the Rest condition (Figure 4).

### Wearing as Compared With Unwearing

During the chewing session under the Wearing condition, significant (*p* < 0.01, one-way repeated-measures ANOVA and Tukey’s test and Dunn’s test) increases in accumulated prefrontal [oxy-Hb] in R-DLPFC (CH 1, 2), R-DLPFC/BA (CH 5), R-DLPFC/FPA (CH 6, 10), L-DLPFC/FPA (CH 8), R-FPA (CH 11), L-FPA (CH 12), R-OFC/FPA (CH 20), and L-OFC/FPA (CH 21) were seen, as compared with that under the Unwearing condition (Figure 4).

### Inter-Correlations Among the Four Independent Variables Related to Increases in Masticatory Score, Burst Duration, and Peak Amplitude for Mm or Ta Between Wearing and Unwearing

Intercorrelations among the four independent variables related to change rates in masticatory score, burst duration, and peak amplitude for Mm or Ta in the Wearing condition, as well as occlusal force, were examined using a bivariate correlation coefficient between the independent variables, with no significant intercorrelations found between them (Tables 4, 5). After the desired variables were entered into the regression model, significant relationships were assessed between dependent variables related to change rates in prefrontal [oxy-Hb] and independent variables related to change rates in masticatory score, burst duration, and ratio of peak amplitudes for Mm or Ta in the Wearing condition, as well as increases in occlusal force.

**TABLE 4:** Bivariate Pearson’s correlation coefficients between independent variables of masticatory score, burst duration [masseter (Mm)], peak amplitude (Mm), and occlusal force in the regression analyses.

**Independent**	**Masticatory**	**Burst**	**Peak**	**Occlusal**
**variables**	**score**	**duration (Mm)**	**amplitude (Mm)**	**force**
Masticatory score		–0.070	–0.098	–0.349
Burst duration (Mm)			0.222	–0.246
Peak amplitude (Mm)				–0.127

**TABLE 5:** Bivariate Pearson’s correlation coefficients between independent variables of masticatory score, burst duration [anterior temporal (Ta)], peak amplitude (Ta), and occlusal force in the regression analyses.

**Independent**	**Masticatory**	**Burst**	**Peak**	**Occlusal**
**variables**	**score**	**duration (Ta)**	**amplitude (Ta)**	**force**
Masticatory score		0.127	−0.209	−0.349
Burst duration (Ta)			−0.467	−0.149
Peak amplitude (Ta)				0.365

### Multiple Linear Regression Analysis of Independent Variables Related to Masticatory Score, Burst Duration, and Peak Amplitude in Mm, as Well as Occlusal Force, and Dependent Variables Related to Prefrontal [oxy-Hb]

**FIGURE 5:**
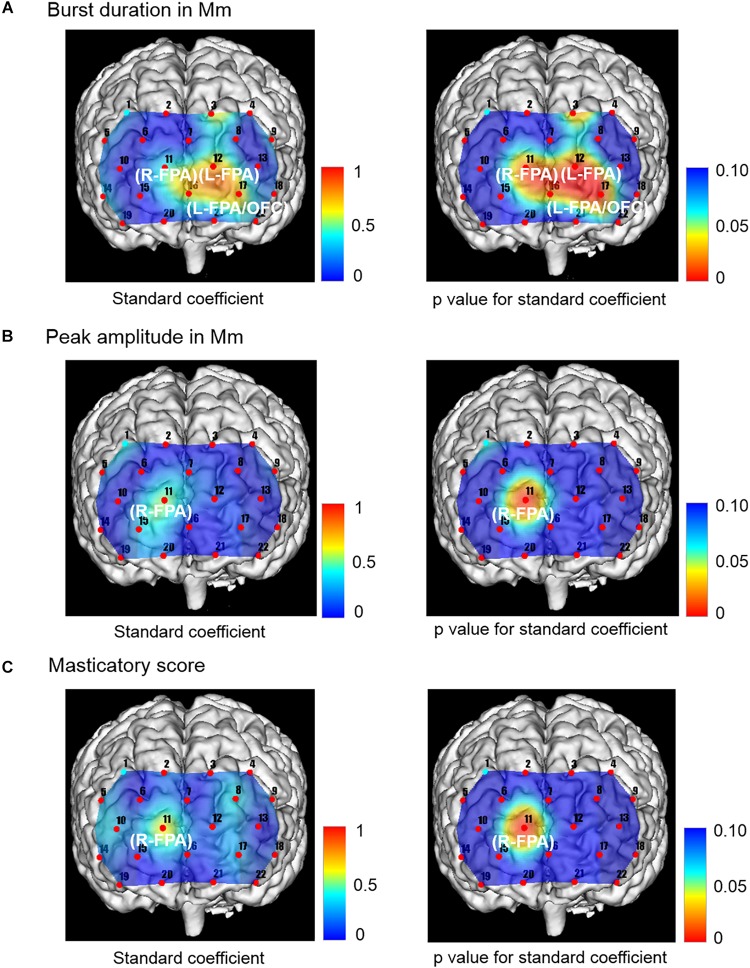
Topographical maps showing standard coefficient and *p* values between independent variables of Mm muscle activities and occlusal force and prefrontal oxygenated hemoglobin concentration [(oxy-Hb)] of the dependent variable in the right and left sides, respectively. **(A)** The change rate in burst duration in the Wearing condition was significantly and positively associated with the change rate in prefrontal [oxy-Hb] during the chewing task in R-FPA (CH 11), L-FPA (CH 12), and L-FPA/OFC (CH 17). **(B)** The change rate in peak amplitude in the Wearing condition was also significantly and positively associated with the change rate in prefrontal [oxy-Hb] during the chewing task in R-FPA (CH 11). **(C)** The change rate in masticatory score in the Wearing condition was significantly and positively associated with the change rate in prefrontal [oxy-Hb] during the chewing task in R-FPA (CH 11).

The change rate in burst duration in the Wearing condition was significantly and positively associated with a change rate in prefrontal [oxy-Hb] during the chewing task in R-FPA (CH 11) (*p* = 0.014, power of performed test with α = 0.050: 0.999), L-FPA (CH 12) (*p* = 0.002, power of performed test with α = 0.050: 0.983), and L-FPA/OFC (CH 17) (*p* = 0.005, power of performed test with α = 0.050: 0.967) (Table 6 and Figure 5A).

**TABLE 6:** Multiple linear regression for association of prefrontal oxygenated hemoglobin concentration [(oxy-Hb)] with masticatory score, burst duration [masseter (Mm)], and peak amplitude (Mm).

**Independent variables**	**Channel (Brodmann area)**	**Coefficients**	**Std. error**	**Standardized coefficients**	***p* value**
Burst duration (Mm)	CH 11 (R-FPA)	2.550	0.876	0.427	0.014*
	CH 12 (L-FPA)	8.231	2.031	0.758	0.002**
	CH 17 (L-FPA/OFC)	9.261	2.654	0.697	0.005**
Peak amplitude (Mm)	CH 11 (R-FPA)	0.417	0.111	0.534	0.003**
Masticatory score	CH 11 (R-FPA)	0.164	0.039	0.631	0.001**

*Significant association of prefrontal [oxy-Hb] (multiple linear regression analysis, *p* < 0.05). **Significant association of prefrontal [oxy-Hb] (multiple linear regression analysis, *p* < 0.01).

The change rate in peak amplitude in the Wearing condition was significantly and positively associated with the change rate in prefrontal [oxy-Hb] during the chewing task in R-FPA (CH 11) (*p* = 0.003, power of performed test with α = 0.050: 0.999) (Table 6 and Figure 5B).

The change rate in masticatory score in the Wearing condition was significantly and positively associated with the change rate in prefrontal [oxy-Hb] during the chewing task in R-FPA (CH 11) (*p* = 0.001, power of performed test with α = 0.050: 0.999) (Table 6 and Figure 5C).

### Multiple Linear Regression Analysis of Independent Variables Related to Burst Duration and Peak Amplitude in Ta, Occlusal Force, and Masticatory Score and Dependent Variables of Prefrontal [oxy-Hb]

**FIGURE 6:**
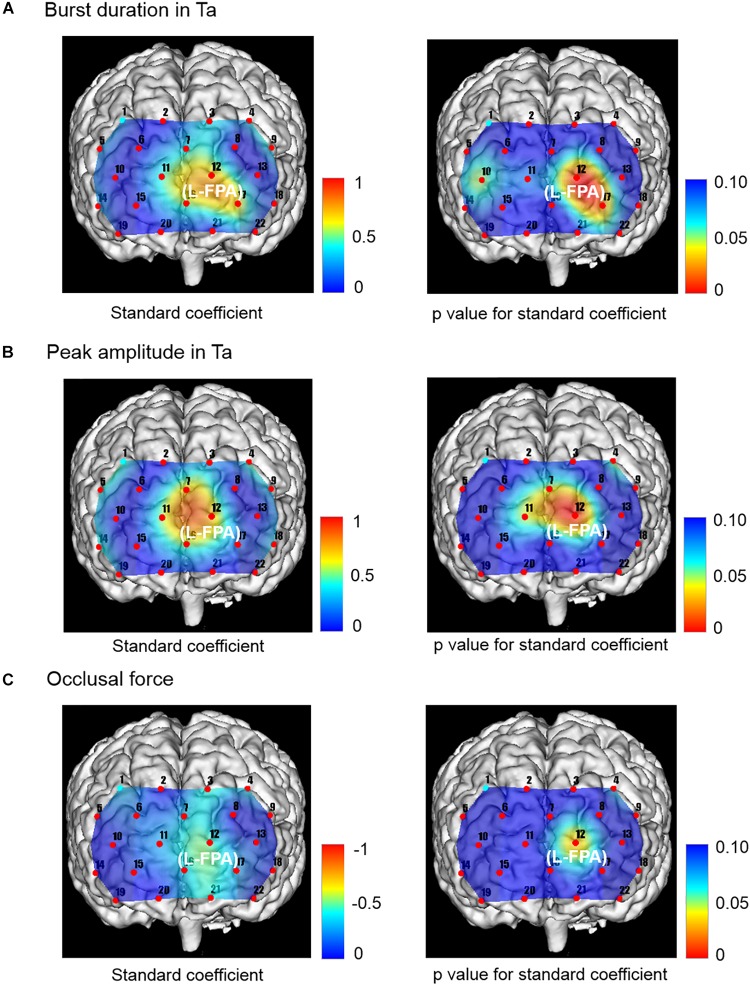
Topographical maps showing standard coefficient and *p* values between independent variables of Ta muscle activities and occlusal force and prefrontal oxygenation hemoglobin concentration [(oxy-Hb)] of the dependent variable in right and left sides, respectively. **(A)** The change rates in burst duration in the Wearing condition were significantly and positively associated with the change rates in prefrontal [oxy-Hb] in L-FPA (CH 12). **(B)** The change rates in peak amplitude in the Wearing condition were significantly and positively associated with the change rate in prefrontal [oxy-Hb] during the chewing task in L-FPA (CH 12). **(C)** The change rate in occlusal force in the Wearing condition was significantly negatively associated with change rate in prefrontal [ oxy-Hb ] during the chewing task in L-FPA (CH 12).

The change rates in burst duration in the Wearing condition were significantly and positively associated with the change rates in prefrontal [oxy-Hb] during the chewing task in L-FPA (CH 12) (*p* = 0.003, power of performed test with α = 0.050: 0.985) (Table 7 and Figure 6A).

**TABLE 7:** Multiple linear regression for association of prefrontal oxygenated hemoglobin concentration [(oxy-Hb)] with burst duration [anterior temporal (Ta)], peak amplitude (Ta), and occlusal force.

**Independent variables**	**Channel (Brodmann area)**	**Coefficients**	**Std. error**	**Standardized coefficients**	***p* value**
Burst duration (Ta)	CH 12 (L-FPA)	6.223	1.654	0.743	0.003**
Peak amplitude (Ta)	CH 12 (L-FPA)	2.896	0.811	0.749	0.004**
Occlusal force	CH 12 (L-FPA)	–1.239	0.513	–0.475	0.034*

*Significant association of prefrontal [oxy-Hb] (multiple linear regression analysis, *p* < 0.05). **Significant association of prefrontal [oxy-Hb] (multiple linear regression analysis, *p* < 0.01).

The change rates in peak amplitude in the Wearing condition were significantly and positively associated with the change rate in prefrontal [oxy-Hb] during the chewing task in L-FPA (CH 12) (*p* = 0.004, power of performed test with α = 0.050: 0.985) (Table 7 and Figure 6B).

The change rate in occlusal force in the Wearing condition was significantly negatively associated with the change rate in prefrontal [oxy-Hb] during the chewing task in L-FPA (CH 12) (*p* = 0.034, power of performed test with α = 0.050: 0.985) (Table 7 and Figure 6C).

## Discussion

This study primarily defined the consistent chewing rhythm formation under Unwearing and Wearing conditions. As compared to the Unwearing condition in our subjects, the Wearing condition resulted in improved masticatory score, occlusal state in regard to force and area, jaw muscle activities in regard to burst duration, area, mean amplitude, and prefrontal activities in DLPFC/FPA/OFC during chewing. In addition, a positive association of the burst duration and peak amplitude of Mm and temporal muscle activities with the prefrontal activities in the Wearing condition was noted as well as masticatory score, while a negative association of occlusal force with prefrontal activities was also noted in the Wearing condition.

Furthermore, the patients investigated in this study presented a masticatory score greater than 80 in subjective evaluations of chewing ability, as well as sufficient occlusal force and masticatory muscle activities under the Wearing condition (Tallgren and Tryde, 1991; Hirai et al., 1994; Miura et al., 1998; Aras et al., 2009). It is therefore conceivable that these positive and negative associations can be considered to reflect the physiological stomatognathic functioning in partially edentulous elderly patients.

### Positive Associations Between Chewing and Prefrontal Activities by Wearing a Denture

The present study found that wearing a denture induces a positive association of burst duration and peak amplitude of jaw muscle activities and masticatory score with prefrontal activities in FPA/OFC. Considering the consistent chewing rhythmicity by the Wearing and Unwearing conditions, the elongated burst duration and increased peak amplitude seen with consistent rhythmical chewing may suggest the enhanced chewing automaticity based on central rhythm and pattern generation in partially edentulous elderly patients (Lund, 1991; Nakamura and Katakura, 1995; Clark, 2015) and associated sensory facilitation by wearing a denture (Morimoto et al., 1989; Hidaka et al., 1999; Grigoriadis and Trulsson, 2018). Furthermore, the prefrontal cortex associated with basal ganglia and sensorimotor cortices may be involved in automatic and habitual control (Takakusaki et al., 2004; Hikosaka and Isoda, 2010; Redgrave et al., 2010) in parallel with the top-down controlling in movement execution (Narayanan and Laubach, 2006; Kamigaki, 2019), sensory percepts (Gilbert and Sigman, 2007; Okamoto et al., 2011; Raos and Savaki, 2017), reward (Horst and Laubach, 2013), and attention (Small et al., 2003).

The prefrontal acceleration caused by wearing a denture may be also supported by the research results showing a positive correlation between chewing ability and jaw closing muscle activities in partially edentulous elderly patients in regard to increased burst duration and peak amplitude, when wearing a denture (Fueki et al., 2008; Kamiya et al., 2016), as well as the finding of chewing-related prefrontal activities (Kamiya et al., 2016; Lin et al., 2016).

Moreover, it has been reported that physical exercise promotes prefrontal physiological and cognitive activities in aged individuals (Bhambhani et al., 2006; Tsujii et al., 2013; Moriya et al., 2016). Such prefrontal positive activation may also be effective to retrieve cognitive activity in aged edentulous individuals by the consolidation of interplay between the hippocampus and prefrontal activities induced by wearing a denture (Preston and Eichenbaum, 2013; Chen et al., 2015; Weilbächer and Gluth, 2016).

Taken together, the finding of elongated burst duration and increased peak amplitude of Mm and temporal muscle activities with consistent chewing rhythmicity by wearing a denture, as well as masticatory score, may be associated with the cognitive prefrontal consolidation in partially edentulous elderly patients, which might occur under usage of good-fitting denture that does not cause unnecessary distraction (Schneider and Shiffrin, 1977; Shiffrin and Schneider, 1977).

### Negative Association of Chewing With Prefrontal Activities Under the Wearing Condition

Our results in this study suggested that wearing a denture induced negative associations of the biomechanical factors in occlusal force with prefrontal activities in FPA.

Findings obtained in previous studies indicate that acceleration of prefrontal cortex activities during usual gait may be induced in older healthy individuals with poor gait speed and/or stride length, as compared with healthy young adults (Mirelman et al., 2017; Hawkins et al., 2018). It is therefore assumed that older adults with poor gait may rely on prefrontal cognition even when moving under a usual gait. Considering this compensative prefrontal participation in old adults, the poor acquisition of biomechanical structure in occlusal force by wearing a denture may be also interpreted as the association of compensative prefrontal recruitments involved in top-down generation of appropriate chewing force in partially edentulous elderly patients.

### Standardization of Jaw Muscle Activities

Standardization of jaw muscle activities during chewing is essential for the critical evaluation of the effects of denture wearing. In the present study, change rates were applied for standardization of chewing-related jaw muscle activities between the Wearing and Unwearing conditions. In addition, in order to define the multiple prefrontal associations with chewing activities, comprehensive standardization was conducted using change rates for prefrontal fNIRS, occlusal force, and mastication score as subjective chewing ability, as well as burst duration and peak amplitude of jaw muscle activities. The results revealed specific prefrontal activation induced by wearing a denture in partially edentulous elderly patients associated with chewing activities.

Ferrario et al. (2004) reported a method for standardization of chewing muscle activities, referred to as jaw muscle EMG activity, under maximum voluntary teeth clenching and used that to compare the effects of two types of denture prostheses. There is no best way to normalize muscle activities, though Halaki and Ginn (2012) suggested obtaining reference values for normalization of EMG activities using the following: (1) maximum activation level during maximum contractions, (2) peak or mean activation level obtained during the task under investigation, (3) activation level during submaximal isometric contractions, and (4) peak-to-peak amplitude of maximum muscle activation. An additional examination may be needed with another method for standardization of jaw muscle activities during chewing performance.

### Measuring fNIRS for Prefrontal Cortex Activities During Chewing Performance

Artifacts in fNIRS can be caused by contractions of temporal muscle blood flow during chewing performance (Schecklmann et al., 2017); thus, scrupulous attention is required to protect the interfusion of muscle artifacts during fNIRS measurements. In this regard, we consider it necessary to comment regarding possible artifact interfusion in the present fNIRS examinations. A previous report presented averaged recording data for masticatory muscle activities and fNIRS obtained during both right- and left-side chewing in order to exclude functional laterality (Kamiya et al., 2016), and the present study also averaged the bilateral fNIRS data. Recently, the dissociated prefrontal cortex activities were presented during chewing performance in patients complaining of oral dysesthesia as compared with healthy controls, even though jaw muscle activities were not different between those two groups (Narita et al., 2019).

In the present study, significant associations of prefrontal fNIRS with jaw muscle activities and occlusal force and masticatory score were noted. Specifically, prefrontal fNIRS was positively associated with burst duration and peak amplitude of the Mm and temporal muscles as well as masticatory score, whereas prefrontal fNIRS was negatively associated with occlusal force (Figures 5, 6). Notably, prefrontal fNIRS was positively associated with burst duration and peak amplitude of temporal muscle activity (Figures 6A,B) and negatively with occlusal force in the same prefrontal localization (Figure 6C). When prefrontal fNIRS data become contaminated by jaw muscle blood flow during chewing performance, these conflicting positive and negative associations cannot simultaneously occur in the same region of the prefrontal cortex and also may not be associated with the bilaterally averaged chewing activities examined in the present study.

The present results also demonstrated separable functional localizations in regard to the associations of prefrontal activities with the peak amplitude of Mm and temporal muscle activities. A prefrontal association of peak amplitude in the Mm muscle was presented in the right FPA, whereas that in temporal muscle was presented in the left FPA (Figures 5B, 6B). In consideration of possible involvement of multiple brain areas in rhythmical chewing, including prefrontal higher cognition, sensorimotor cortices, and subcortical areas (Quintero et al., 2013; Lin et al., 2016, 2017), an additional study that utilizes whole-brain network analysis may be necessary to more appropriately interpret functional laterality in regard to the association of the prefrontal cortex with chewing activities in partially edentulous elderly patients when wearing a denture.

## Conclusion

This study was conducted to clarify the efficacy of wearing a denture in partially edentulous elderly patients from the viewpoint of associations between prefrontal and chewing activities in regard to chewing ability, occlusal force, and chewing muscle activities. Our results show that wearing a denture improves prefrontal activity, occlusal state, chewing muscle activities, and masticatory score, as compared with not wearing one. Additionally, the prefrontal activities were positively associated with burst duration and peak amplitude of Mm and temporal muscle activities and masticatory score by wearing a denture. In contrast, the prefrontal activities were negatively associated with occlusal force by wearing a denture.

In consideration of prefrontal cognitive activation caused by physical exercise (Bhambhani et al., 2006; Tsujii et al., 2013; Moriya et al., 2016), the positive relationships between the temporal burst duration and peak amplitude in Mm and temporal muscle activities, as well as masticatory score, and the associated prefrontal activation induced by wearing a denture may be considered as the prefrontal consolidation under the condition of the enhanced chewing automaticity in partially edentulous elderly patients. On the other hand, the negative relationships between the occlusal force and the prefrontal activations shown by wearing a denture could be interpreted as the prefrontal compensative participation in order to precisely generate the biomechanical occlusal force during chewing performance in partially edentulous elderly patients (Spraker et al., 2009; Derosière et al., 2014).

Findings obtained in this study warrant further investigations for the better understanding of oral neurorehabilitation based on Eichner’s intermaxillary tooth contact classification in the individual elderly patient.

## Data Availability Statement

The datasets generated for this study are available on request to the corresponding author.

## Ethics Statement

The present study protocol was approved by the Ethics Committee of Nihon University School of Dentistry at Matsudo (EC 14-13-010-1), and all patients provided written informed consent prior to participation.

## Author Contributions

NN designed the study and wrote the manuscript. KK collected the data. TI and KK analyzed the obtained data. SI, MO, TU, IK, and KS contributed to the data interpretation.

## Conflict of Interest

TU and IK were employed by company Dental Support Co. Ltd. and have no potential conflicts of interest with respect to the research, authorship, and/or publication of this article. The remaining authors declare that the research was conducted in the absence of any commercial or financial relationships that could be construed as a potential conflict of interest.

## Abbreviations

AD: anterior digastric
BA: pars triangularis Broca’s area
DLPFC: dorsolateral prefrontal cortex
EMG: electromyography
FPA: frontopolar area
fMRI: functional magnetic resonance imaging
fNIRS: functional near-infrared spectroscopy
IPG: inferior prefrontal gyrus
Mm: masseter
OFC: orbitofrontal cortex
Ta: anterior temporal.
