# Supplementary material for: Prefrontal Consolidation and Compensation as a Function of Wearing Denture in Partially Edentulous Elderly Patients
Source: Front Aging Neurosci. 2020 Jan 31;11:375. doi: 10.3389/fnagi.2019.00375 (PMC7005254; doi:10.3389/fnagi.2019.00375)
Supplement: Supplementary file 1 [file Table_1.docx]

Supplementary Table 1 Number of remaining teeth in right and left sides in 16 partially edentulous patients

| Number of remaining teeth | Left side | | Right side | |  |
| --- | --- | --- | --- | --- | --- |
|  | Mean | SD | Mean | SD | p value |
|  | 9.062 | 1.691 | 8.812 | 1.939 | 0.554 |

There were no significant (paired *t*-test) differences in regard to the number of remaining teeth between right and left sides in 16 partially edentulous patients.
