# Supplementary material for: Prefrontal Consolidation and Compensation as a Function of Wearing Denture in Partially Edentulous Elderly Patients
Source: Front Aging Neurosci. 2020 Jan 31;11:375. doi: 10.3389/fnagi.2019.00375 (PMC7005254; doi:10.3389/fnagi.2019.00375)
Supplement: Supplementary file 2 [file Table_2.docx]

Supplementary Table 2 Prefrontal [oxy-Hb] during right- and left-side chewing under Unwearing conditions.

| Prefrontal [oxy-Hb] (mMmm) | Left side chewing | | Right side chewing | |  |
| --- | --- | --- | --- | --- | --- |
|  | Mean | SD | Mean | SD | p value |
| CH 1 | 0.442 | 2.891 | 0.397 | 2.836 | 0.954 |
| CH 2 | 0.120 | 2.886 | 0.167 | 2.473 | 0.929 |
| CH 3 | 0.299 | 3.202 | 0.359 | 2.549 | 0.917 |
| CH 4 | 0.175 | 2.440 | 0.533 | 2.247 | 0.496 |
| CH 5 | 1.339 | 2.400 | 1.300 | 2.572 | 0.958 |
| CH 6 | 0.460 | 3.236 | 0.656 | 2.374 | 0.747 |
| CH 7 | 0.240 | 2.872 | 0.108 | 2.445 | 0.811 |
| CH 8 | 0.535 | 3.094 | 0.488 | 2.397 | 0.93 |
| CH 9 | 1.445 | 2.760 | 1.923 | 3.297 | 0.51 |
| CH 10 | 0.887 | 3.895 | 1.017 | 2.922 | 0.846 |
| CH 11 | 0.463 | 3.292 | -0.089 | 2.336 | 0.441 |
| CH 12 | 0.171 | 3.639 | -0.238 | 3.205 | 0.563 |
| CH 13 | 1.196 | 3.032 | 1.339 | 4.022 | 0.851 |
| CH 14 | 1.397 | 4.048 | 0.879 | 3.798 | 0.460 |
| CH 15 | 0.253 | 4.409 | 0.105 | 3.148 | 0.870 |
| CH 16 | 0.535 | 4.700 | 0.027 | 2.951 | 0.564 |
| CH 17 | 0.320 | 4.776 | 0.193 | 3.749 | 0.885 |
| CH 18 | 1.345 | 3.874 | 0.111 | 4.210 | 0.128 |
| CH 19 | 0.217 | 4.282 | -0.272 | 2.810 | 0.554 |
| CH 20 | -0.734 | 4.366 | -1.020 | 2.740 | 0.698 |
| CH 21 | -0.344 | 4.455 | -0.211 | 3.426 | 0.864 |
| CH 22 | 0.738 | 4.520 | 0.016 | 3.985 | 0.435 |

There were no significant (paired *t*-test) differences in regard to the prefrontal [oxy-Hb] values between right- and left-side chewing under the Unwearing conditions.
