# Supplementary material for: Prefrontal Consolidation and Compensation as a Function of Wearing Denture in Partially Edentulous Elderly Patients
Source: Front Aging Neurosci. 2020 Jan 31;11:375. doi: 10.3389/fnagi.2019.00375 (PMC7005254; doi:10.3389/fnagi.2019.00375)
Supplement: Supplementary file 3 [file Table_3.docx]

Supplementary Table 3 Prefrontal [oxy-Hb] during right- and left-side chewing under Wearing conditions.

| Prefrontal [oxy-Hb] (mMmm) | Left side chewing | | Right side chewing | |  |
| --- | --- | --- | --- | --- | --- |
|  | Mean | SD | Mean | SD | p value |
| CH 1 | 2.475 | 2.966 | 1.725 | 1.891 | 0.141 |
| CH 2 | 1.859 | 2.585 | 1.116 | 2.237 | 0.125 |
| CH 3 | 1.526 | 2.504 | 0.848 | 2.176 | 0.138 |
| CH 4 | 1.602 | 2.208 | 1.305 | 2.031 | 0.607 |
| CH 5 | 2.896 | 2.758 | 2.407 | 2.171 | 0.436 |
| CH 6 | 3.589 | 2.674 | 2.344 | 2.422 | 0.052 |
| CH 7 | 1.984 | 2.551 | 0.744 | 2.506 | 0.215 |
| CH 8 | 2.499 | 2.145 | 1.879 | 2.501 | 0.297 |
| CH 9 | 2.213 | 2.635 | 2.425 | 2.307 | 0.741 |
| CH 10 | 3.078 | 2.649 | 3.081 | 2.873 | 0.996 |
| CH 11 | 2.248 | 2.444 | 1.476 | 3.258 | 0.218 |
| CH 12 | 2.445 | 3.407 | 1.715 | 3.870 | 0.355 |
| CH 13 | 2.618 | 2.649 | 3.195 | 3.009 | 0.377 |
| CH 14 | 3.045 | 4.333 | 4.135 | 5.161 | 0.377 |
| CH 15 | 2.065 | 3.614 | 2.776 | 4.820 | 0.439 |
| CH 16 | 1.829 | 3.339 | 1.339 | 3.280 | 0.291 |
| CH 17 | 2.359 | 3.374 | 1.954 | 3.279 | 0.547 |
| CH 18 | 1.757 | 2.932 | 2.638 | 2.970 | 0.373 |
| CH 19 | 1.231 | 3.246 | 2.013 | 3.625 | 0.458 |
| CH 20 | 0.550 | 3.354 | 1.384 | 3.620 | 0.262 |
| CH 21 | 1.401 | 3.001 | 1.554 | 3.887 | 0.811 |
| CH 22 | 2.532 | 2.942 | 2.335 | 3.454 | 0.802 |

There were no significant (paired *t*-test) differences in regard to the prefrontal [oxy-Hb] values between right- and left-side chewing under the Wearing conditions.
