# Supplementary material for: Prefrontal Consolidation and Compensation as a Function of Wearing Denture in Partially Edentulous Elderly Patients
Source: Front Aging Neurosci. 2020 Jan 31;11:375. doi: 10.3389/fnagi.2019.00375 (PMC7005254; doi:10.3389/fnagi.2019.00375)
Supplement: Supplementary file 4 [file Table_4.docx]

|  | Supplementary Table 4 Masticatory muscle EMG activities during right- and left-side chewing under Unwearing conditions. | | | | | | |
| --- | --- | --- | --- | --- | --- | --- | --- |
| Masticatory muscle EMG activities | |  | Left side chewing | | Right side chewing | |  |
|  |  |  | Mean | SD | Mean | SD | p value |
| Number of chewing strokes | | Mm | 63.437 | 13.381 | 63.125 | 14.009 | **0.898** |
| Cycle duration (ms) | | AD | 802.750 | 197.841 | 728.000 | 134.888 | **0.193** |
| Burst duration (ms) | | Mm | 311.185 | 87.826 | 301.901 | 103.078 | 0.649 |
|  | | Ta | 281.013 | 73.992 | 283.359 | 73.232 | 0.873 |
| Inter-burst duration (ms) | | Mm | 522.546 | 105.337 | 548.948 | 119.623 | **0.700** |
|  | | Ta | 553.555 | 99.331 | 569.654 | 118.384 | 0.462 |
| Area (mV･s) | | Mm | 0.014 | 0.011 | 0.011 | 0.006 | **0.348** |
|  | | Ta | 0.011 | 0.006 | 0.009 | 0.004 | 0.201 |
| Mean amplitude (mV) | | Mm | 0.048 | 0.036 | 0.041 | 0.032 | 0.495 |
|  | | Ta | 0.043 | 0.025 | 0.036 | 0.021 | 0.217 |
| Peak amplitude (mV) | | Mm | 0.116 | 0.081 | 0.089 | 0.061 | **0.831** |
|  | | Ta | 0.073 | 0.043 | 0.058 | 0.036 | 0.097 |

Paired *t*-test is indicated in normal font, and Wilcoxon’s signed rank test is indicated in bold font. There were no significant (paired *t*-test and Wilcoxon’s signed rank test) differences in regard to the masticatory muscle EMG activities between right- and left-side chewing under Unwearing conditions.
